# Achondroplasia among ancient populations of mesoamerica and South America: Iconographic and Archaeological Evidence.

**Published:** 2012-09-30

**Authors:** Carlos A Rodríguez, Carolina Isaza, Harry Pachajoa

**Affiliations:** a Faculty of Integrated Arts, Universidad del Valle. E-mail: carlos.a.rodriguez@correounivalle.edu.co; b Faculty of Health. Universidad del Valle. E-mail: carolinaisa@cable.net.co; c Faculty of Health. Universidad del Valle. Faculty of Health Sciences, Universidad Icesi. E-mail: hmpachajoa@icesi.edu.co

**Keywords:** Achondroplasia, genetics, archaeology, dwarfism

## Abstract

**Introduction:**

Achondroplasia is the most frequent form of short-limb dwarfism. Affected individuals exhibit short stature caused by rhizomelic shortening of the limbs, characteristic facies with frontal bossing and mid-face hypoplasia, genu varum, and trident hand. Although the etiology of this disease was reported in 1994, evidence of this disease in ancient populations has been found in populations of ancient Egypt (2500 BC) and it has been documented in ancient American populations.

**Objective:**

To analyze the presence of individuals with achondroplasia in the Mayan state society of Mexico and Guatemala, during the Classical (100- 950 AC ) and Post-Classical (950 - 1519 AC ) periods; likewise, in the hierarchical-chieftain society of Tumaco-la Tolita (300 BC - 600 AC ) from the Colombia-Ecuador Pacific coast, and the Moche state society (100 - 600 AC ) from the northern coast of Peru.

**Methods:**

Iconographic and clinical-morphological studies of some of the most important artistic representations of individuals of short stature in these three cultures.

**Conclusion:**

We present the hypothesis that the individuals with short stature were somehow associated with the political and religious power elite.

## Abstract (es)

**Introducción:**

La acondroplasia es la forma más frecuente de enanismo. Los individuos presentan acortamiento rizomélico de las extremidades, facies característica con prominencia frontal, hipoplasia media de la cara, genu varo, y la mano tridente. Aunque la etiología de esta enfermedad fue reportada en 1994, evidencia de la existencia esta enfermedad se ha encontrado en poblaciones del antiguo Egipto (2500 aC) y ha sido documentada en poblaciones americanas antiguas.

**Objetivo:**

Analizar la presencia de personas con acondroplasia en la sociedad del estado maya de México y Guatemala, durante el período clásico (100- 950 aC ) y postclásico ( 950 a 1519 aC ), del mismo modo, en la sociedad de Tumaco-la Tolita (300 aC - 600 dC) de la costa del Pacífico entre Colombia y Ecuador, y el Estado y la sociedad Moche (100- 600 aC ) de la costa norte del Perú.

**Métodos:**

Se realizaron estudios iconográficos y clínico morfológico de algunas de las representaciones artísticas más importantes de las personas de talla baja en estas tres culturas.

**Conclusiones:**

Se presenta la hipótesis de que los individuos con talla baja se asociaban de alguna manera con la élite del poder político y religioso.

## Introduction

Achondroplasia (MIM: 100800) is the most frequent form of short-limb dwarfism. Affected individuals exhibit short stature caused by rhizomelic shortening of the limbs, characteristic facies with frontal bossing and mid-face hypoplasia, genu varum, and trident hand. It is an autosomal dominant disorder; most cases are sporadic, the result of a de novo mutation (mutation in the fibroblast growth factor receptor-3 gene, FGFR3)^1^, and its prevalence has been estimated at 1 in every 16,000 births to 1 in every 35,000 births and it is estimated that currently there are 65,000 individuals in the world with this disease^2^.

Although the etiology of this disease was reported in 1994, evidence of this disease in ancient populations has been found in populations of ancient Egypt (2500 BC)^3^ ^,^ ^4^ and it has been documented in ancient American populations, based on archaeological sources, for at least since 2300 years ago ^5^ ^-^ ^7^. In effect, in visual languages of distinct pre-Colombian cultures, in support as diverse as ceramic artifacts, lithic artifacts, and amate paper, individuals of short stature are depicted, associated with the political and religious power elite. These depictions appear both in complex chieftain cultures, as in early state societies, which existed in Latin America between 300 BC and 1500 AC.

Within this article, we analyze the presence of individuals with achondroplasia in the Mayan state society of Mexico and Guatemala, during the Classical (100-950 AC) and Post-Classical (950 - 1519 AC) periods; likewise, in the hierarchical-chieftain society of Tumaco-la Tolita (300 BC - 600 AC) from the Colombia-Ecuador Pacific coast, and the Moche state society (100 - 600 AC) from the northern coast of Peru. We conducted iconographic and clinical-morphological studies of some of the most important artistic representations of individuals of short stature in these three cultures. We also state the hypothesis that the individuals with short stature were somehow associated with the political and religious power elite. 

## Achondroplasia in the tumaco-la tolita culture

The Tumaco-La Tolita culture inhabited the Colombia-Ecuador Pacific coast region between 300 BC and 600 AC. Their findings have been reported from the Esmeraldas River to the south of the province of Esmeraldas in Ecuador to the San Juan River in the department of Valle del Cauca in Colombia. Among the nearly 20 representations of genetic disease and congenital malformations detected in the ceramic artifacts from the populations of the Tumaco-La Tolita culture^5^ ^-^ ^7^, based on medical and iconographic evaluation, achondroplasia appears as a relatively frequent mono-genic alteration^8^ ^,^ ^9^.

**Figure 1:**
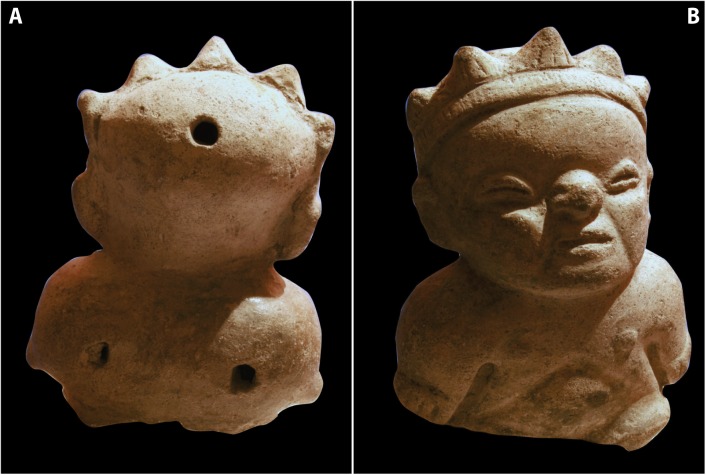
A. Character with achondroplasia, molded ceramic technique. B. On the posterior upper part of the piece we can see the mouthpiece on the center of the head and the two holes on the arms, characteristic of wind instruments denominated whistles.

Characters with short stature were personified in ceramic artifacts, using the molding technique. As noted in Figure 1A-B an individual with some type of crown is depicted. The anthropomorphic piece was used as a musical instrument, specifically, a double whistle, as can be inferred by three holes on the upper part. The phenotypical characteristics pertain to achondroplasia, e.g., frontal bossing, depressed nasal bridge, short nose because of hypoplasia of nasal bones, and shortened upper and lower extremities are clearly shown in these representations. The headdress in the shape of a crown, in a clear reference to solar rays, as well as the function of the piece associated with music, are two important elements that, in our concept, suggest the relationship of these achondroplasic individuals with shamanistic activities. 

**Figure 2:**
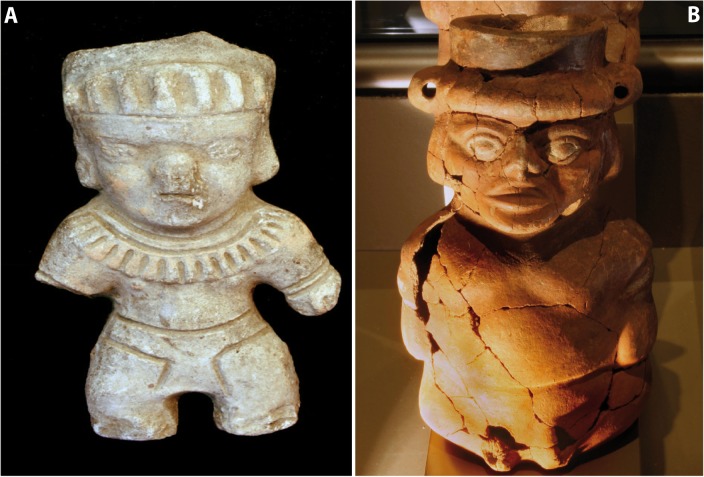
A. Ceramic figurine representing an achondroplasic individual. B. Detail of one of the individuals, note the big head and the shortened arms and legs, phenotypical characteristics of individuals with achondroplasia.

Another very suggestive image of an achondroplasic individual is shown in (Figure 2A), which depicts the full-body character with a loincloth. This individual is adorned with some type of hat and a collar that practically covers the whole chest, attributes of the power elites. 

## Achondroplasia in the Moche culture

During the first 600 years of our era in the Moche state society from the Peruvian coast, small individuals, of short stature and with different features from the rest of the population, appear to have been very important. They were represented in ceramic artifacts, both as human figures, and as part of visual thematic narratives painted on ceramic vases known as ''stirrup spout bottles''^10^.

Among the 1,137 ceramic pieces found in the offerings site for the Señor de Sipán (Lord of Sipán)^10^, several vessels were found with human shapes depicting achondroplasic individuals, which were placed as funerary trousseau. Nine of these sculptures are currently on display in a special showcase of the Royal Tombs of Sipán Museum. All the sculptures have eyes painted white and a headdress upon their heads formed by a rolled cloth in the shape of a ''turban'' (head ring) (Fig. 2B). Some researchers consider that among the Moche, the headdresses might have been the most important distinction of social status or of the ritual function of the individual depicted^10^.

But small individuals were not only materialized in Moche art; they were also present on visual thematic narratives painted with different colors on stirrup spout bottles. These types of complex designs represent a great number of scenes of everyday life, as well as of the cosmos and the gods and depict some wonderful examples of Moche visual art. 

**Figure 3:**
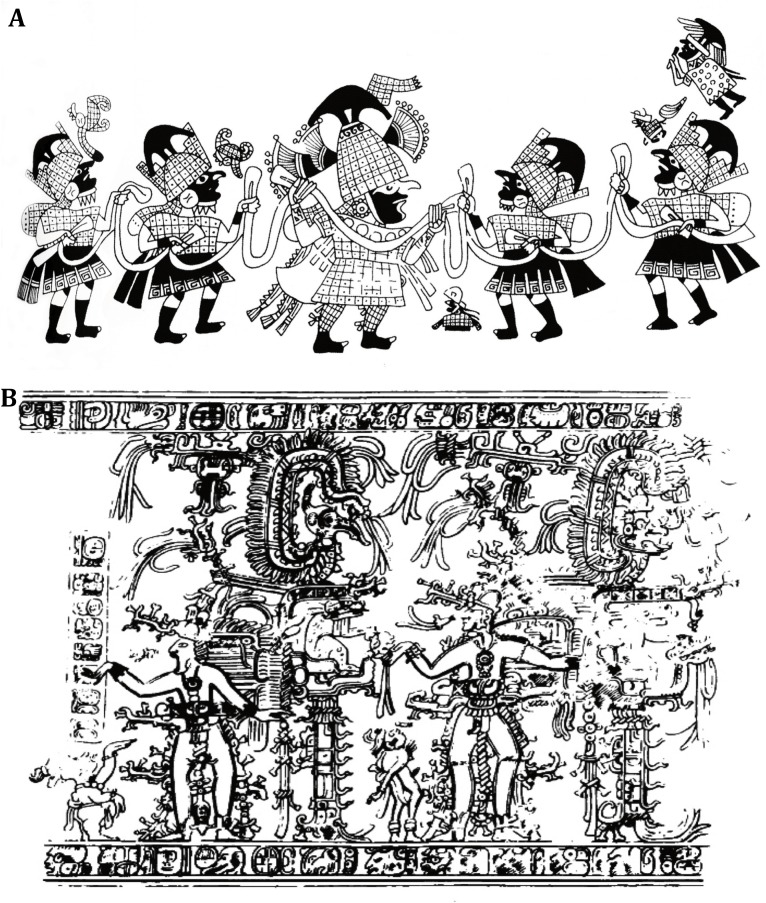
A. Scene of the dance of warriors (taken from reference 10). B. Cylinder vase from Yalloch (taken from reference 11).

We wish to point out a scene denominated the ''rope dance''. A group of warriors with sumptuous garbs and headdresses, where there is the figure of a leader in the center of the composition and another four warriors to his sides; they are all holding some type of rope. They are all wearing decorated helmets with a half moon on the central part. Three more individuals are part of this representation; two of them are achondroplasic with similar vestments as those worn by the warriors, but with less sumptuous headdresses. The first is between the central character and the warrior on the right. The second is on the upper right next to an individual appearing to be playing a flute (Shaman?). Unlike the achondroplasic individual on the lower part, this character has a complete body and has an unidentified object in his hand (Fig. 3A). Within the general context of the depiction, these two small individuals seem to be an important part of the mythical composition associated with power. 

## Ppuses in the society of the ancient mayas

Among the state societies of the ancient Mayas, achondroplasia seems to have been common; judging from recurrent depictions in art. Individuals with these physical characteristics were sculpted in high relief, both on the commemorative stelae of the governors, as in whistles and small ceramic thrones; also on jade pectorals and in everyday scenes and/or rituals carried out with polychrome paint on ceremonial cylindrical ceramic vases^11^.

Recent iconographic studies suggest that individuals of low stature could have fulfilled a great amount of social roles; among them, serving as symbols of liminality. In effect, the monumental representations of achondroplasic individuals could symbolize the two existential planes, according the Mayan conception of time and its integration and dialectic complement of the natural and the supernatural^12^.

Hereinafter, we illustrate three figures that, in our concept, suggest the importance achondroplasic individuals had in classical and late classical Mayan art. The first, elaborated in ceramic, comes from excavations conducted in 1957 in Pre-Hispanic Mayan burial grounds in the isle of Jaina, Campeche (Mexico). This is a ceramic figurine, which on its head a sumptuous turban. Morphometric measurements undoubtedly correspond to an achondroplasic adult male (Fig. 3B). The symbolism of the headdress is associated with the sun, a vital element of life on the earth. According to Seler (1904) on the 4.Olin day and during eclipses, animals and people who could have affinity with the solar star were sacrificed to avoid endangering the sun. Dwarfs were among the individuals offered for sacrifice^11^.

The second representation is a polychrome thematic narrative depicted on the external surface of a cylindrical ceremonial vase from the Classical Period, coming from a burial in a Mayan chulltun in Yalloch, Petén (Guatemala) (Fig. 3B). 

**Figure 4:**
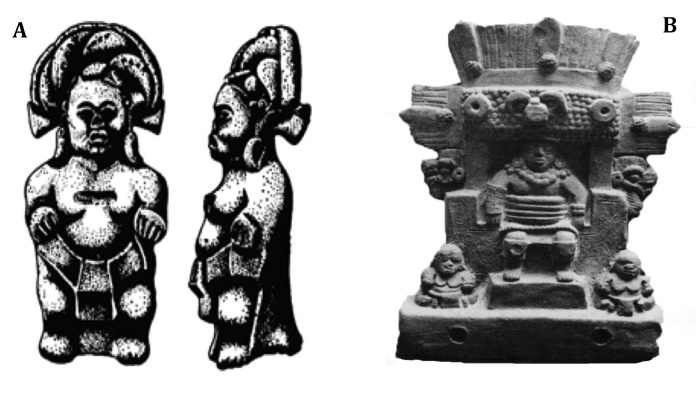
A. Ppuse with elongated ears in chili shape and a headdress as adornment, with red paint covering the whole body (taken from reference13).B. Mayan governor seated at his throne, accompanied by two dwarfs.

The scene, which most likely represents the corn ritual, includes several standing human figures, zoomorphic images, religious symbols, and hieroglyphics. It is divided into two parts in which there are two central individuals performing some type of religious ritual, related to song and dance. On the lower left end, which is of our interest, there is the representation of a standing achondroplasic subject, who is talking to the central individual and extending him his left hand. The central individual appears with a headdress and open mouth, possibly singing. The position of the Ppuse, on the lower part and in the general context of the scene, suggests its importance in the ceremony (Fig. 4A)^13^.

Finally, we refer to a ceramic tablet from the, already mentioned, burial grounds of Jaina, which is currently in the collection of the National History and Anthropology Museum in Mexico. As can be seen, this is a three-dimensional depiction of a governor dressed for a ball game. He is seated at a throne and accompanied by two dwarfs located on the lower part, one on each side of his feet. In this case, both small individuals fulfill the function of companions for the central character in an activity, which, like the ball game, had great symbolic importance in classical Mayan society (Fig. 4B). This function of achondroplasic individuals as companions of political and religious leaders was also fulfilled in the voyage after death. 

## Conclusions

Studies of the last 30 years in the fields of archaeology, medical archaeology, and ichonography have demonstrated that achondroplasic individuals were considered special beings in pre-Colombian societies of Meso-America and South America, where they fulfilled a great many functions in everyday life, as well as in different rites associated with the divinities. Among the ancient Mayas, achondroplasic individuals worked in state administration collecting taxes and controlling the quality of products; they were also part of the group of those working in culture as artists (musicians). They could serve governors, being part of their followers and accompanying them in diverse public and religious activities. They are also represented scenes of song and dance, associated to shaman activities^14^. For its part, in the society of Tumaco-La Tolita individuals of short stature frequently participated in feasts and ritualistic activities associated with shamanism. Also, in Moche art, achondroplasic individuals recurrently appear associated to rites in which the governors and the gods participated.

These small giants, through their quality as unique beings, served as a connection with the world of the dead and were believed to have supernatural powers; a condition that still persists in many indigenous communities in the American continent, as with the current Amazonian Uitotos and the Muiname, for whom power is seen as that which is below and not as that which is above. 
